# Dietary total antioxidant capacity and risk of stroke: a systematic review and dose–response meta-analysis of observational studies

**DOI:** 10.3389/fnut.2024.1451386

**Published:** 2024-09-19

**Authors:** Yiqian Huang, Yajun Ni, Lin Yu, Long Shu, Qin Zhu, Xingzhen He

**Affiliations:** ^1^Department of Anesthesia Operation, Zhejiang Hospital, Hangzhou, Zhejiang, China; ^2^Department of Nutrition, Zhejiang Hospital, Hangzhou, Zhejiang, China; ^3^Department of Digestion, Zhejiang Hospital, Hangzhou, Zhejiang, China

**Keywords:** dietary total antioxidant capacity, stroke, systematic review, dose–response meta-analysis, observational studies

## Abstract

**Background:**

Previous studies have reported the association between dietary total antioxidant capacity (TAC) and risk of stroke, but these findings have been inconsistent. We therefore performed this systematic review and dose–response meta-analysis of observational studies to evaluate the association between dietary TAC and risk of stroke.

**Methods:**

A systematic literature search was carried out through PubMed, ISI Web of Science, EBSCO, Scopus and China National Knowledge Infrastructure (CNKI) databases, to find the relevant articles published up to 31 May, 2024. Random-effects or fixed-effects models were used to pool the relative risks (RRs) and their 95% confidence intervals (CIs) where appropriate. Heterogeneity across studies were determined using the Cochran’s *Q* test and I-square (*I*^2^) statistics.

**Results:**

Eight observational studies (six cohort and two case–control studies) were included in the final analysis. The pooled results showed that higher intake of dietary TAC was associated with a lower risk of stroke (RR = 0.88; 95%CI: 0.81–0.95, *p* = 0.002). Additionally, dose–response analysis of cohort studies demonstrated a linear association between dietary TAC intake and risk of stroke (RR = 0.994; 95%CI: 0.990–0.999, *P*_non-linearity_ = 0.329, *P*_dose–response_ = 0.014). Subgroup analyses showed the inverse association between dietary TAC intake and risk of stroke in the studies with mean age < 50 (RR = 0.82, 95%CI: 0.67–0.99, *p* = 0.044), and there was no evidence of heterogeneity (*p =* 0.360; *I*^2^ = 0.0%).

**Conclusion:**

Our findings indicated that higher intake of dietary TAC was inversely associated with the risk of stroke. Future studies in particular of longitudinal design are needed to confirm this inverse relationship.

**Systematic review registration:**

https://www.crd.york.ac.uk/prospero/display_record.php?ID=CRD42024547706.

## Introduction

Stroke is a prevalent global disease, with an estimated 12.2 millions new cases and 101 million prevalent cases in 2019 (1). It ranks as the second leading cause of death and the third leading cause of death and disability combined worldwide, posing a serious threats to public health (2). The Global Burden of Disease Study (GBD) 2017 estimated that stroke-related deaths in China reached approximately 2 million in 2017, making stroke as a leading cause of death (3). The well-established risk factors for stroke included smoking, physical inactivity, obesity, hypertension, diabetes, cardiovascular disease and unhealthy diet (4). Therefore, the implementation of effective prevention strategies is essential to mitigate the burden of this disease.

Over the past few decades, it has been suggested that dietary factors have an essential role in preventing stroke (5). Notably, evidence has shown that some kinds of dietary patterns, minerals and vitamins play an important role in preventing various chronic diseases, such as cardiovascular diseases and diabetes (6, 7). However, there was not sufficient literature examining the benefits of dietary antioxidants intake on cardiovascular diseases, particularly stroke. A previous meta-analysis found that increased consumption of fruit and vegetables was associated with a reduced risk of stroke (8). Researchers hypothesized that phytochemicals and vitamins with antioxidant properties present in fruit and vegetables might play a pivotal role in this favorable effect (9). Indeed, dietary antioxidants have been known to scavenge free radicals and reduce oxidative stress (10). Previous epidemiological studies have mainly focused on the effects of the intake of individual antioxidants or a group of antioxidants on the risk of stroke (11–13). For example, in an earlier meta-analysis of prospective studies, dietary intake of vitamin C was significantly associated with a lower risk of stroke (14). However, due to the synergistic effects of different dietary antioxidants, an assessment of individual antioxidant intake May not fully reflect the total antioxidant capacity of the diet (15). To consider this, dietary total antioxidant capacity (TAC) has been developed as a suitable tool for studying the potential beneficial effects of the overall dietary antioxidants, taking into account the cumulative/synergistic effects of various antioxidants and their interactions with each other (16).

Recently, dietary TAC has garnered considerable attention in nutritional research (17). Accumulating evidence suggests that dietary TAC is inversely associated with adverse health outcomes, such as cardiovascular diseases, cancer and mortality (16, 18, 19). To date, only a few epidemiological studies have evaluated the association between dietary TAC and risk of stroke (9, 20–26). However, findings of these previous studies were inconsistent. While some studies reported an inverse association between dietary TAC and risk of stroke (21, 23), others found a non-significant association (24–26). Furthermore, to our best knowledge, no systematic review and dose–response meta-analysis has so far been conducted to assess the association between dietary TAC and risk of stroke. Therefore, to ascertain the association between dietary TAC and risk of stroke, we conducted a systematic review and dose–response meta-analysis to summarize the findings from observational studies published up to May 2024.

## Methods

### Protocol and registration

This study complied with the Preferred Reporting Items for Systematic Reviews and Meta-Analyses (PRISMA) guidelines (27). The protocol of this meta-analysis has been registered in the International Prospective Register of Systematic reviews (PROSPERO), and its registration number is CRD42024547706.

### Search strategy

A systematic search using PubMed, ISI Web of Science, EBSCO, Scopus, and CNKI databases was performed to find the studies that evaluated the association between dietary TAC and stroke up to 31 May, 2024. The search strategy included the following keywords: “dietary antioxidant capacity,” “dietary total antioxidant capacity,” “dietary TAC,” “non-enzymatic antioxidant capacity,” “dietary antioxidant index,” “antioxidant capacity of diet” and “stroke.” Moreover, hand-searching from reference lists of all relevant articles, previous reviews and meta-analyses was performed to identify relevant studies. Meanwhile, unpublished studies or gray literature were not included in this meta-analysis. The complete search strategy could be found in the Supplementary Table S1. Two authors (Y.-Q.H and L.S.) independently screened and crosschecked each article from the literature search, and a third author (X.-Z.H) was consulted to resolve any discrepancies.

### Study selection

In the initial search, two authors (Y.-Q.H and X.-Z.H.) independently screened the titles and abstracts of the retrieved articles and excluded duplicates and irrelevant articles. Then, the full-text versions of the articles were reviewed basing on the inclusion and exclusion criteria of the current systematic review and meta-analysis. To be included in our analyses, studies must meet all of the following eligibility criteria: (1) observational studies, e.g., cohort, case–control or cross-sectional studies; (2) those studies were published in English or Chinese languages; (3) the exposure of interest was dietary TAC; (4) the outcome of interest was stroke, including any fatal/non-fatal ischemic stroke, hemorrhagic stroke, or other cerebrovascular accidents; (5) studies providing the relative risks (RRs), odds ratios (ORs) and hazards ratios (HRs) along with their corresponding 95% confidence intervals (CIs) of stroke (or sufficient data to calculate them); (6) If the original data in the retrieved studies lacked sufficient detail, the corresponding author of this study would be contacted by email twice. Besides, studies were excluded if they met one of the following criteria: (1) animal, cell culture, and *in vitro* studies; (2) non-observational studies, including conference abstracts, reviews, editorials, case reports, book chapters, and letters; (3) did not provide the HRs, RRs or ORs with corresponding 95% CIs; (4): the exposure of interest was single antioxidant, such as vitamin C; (5) Irrelevant articles. In case of multiple published reports on the same dataset, we selected the most recent study; Otherwise, the one with the most number of cases was selected. When results of a study for men and women were reported separately, we treated each analysis as a separate study.

### Data extraction

Two independent authors (L.Y. and Y.-J.N) independently extracted the following information from all selected article: the first author’s last name, publication year, study design, study region, sample size, number of participants and stroke cases, mean age/age range, duration of follow-up in cohort studies, methods used for assessing dietary TAC, confounding variables adjusted in the multivariate analyses, and reported risk estimates with their corresponding 95% CIs of stroke across categories of dietary TAC.

### Quality assessment

The authors (L.S. and Q.Z) independently assessed the quality of included studies using the Newcastle-Ottawa Scale(NOS), which was designed for non-randomized studies (28). This scale is composed of eight items in three domains: study selection, comparability of participants, and assessment of outcome/exposure of interest, with a maximum score of 9. Studies with NOS scores ≥7 points were classified as high quality (29). Any discrepancies between two authors were resolved by a third author to reach a consensus.

### Definition of dietary TAC

Dietary TAC, also known as the non-enzymatic antioxidant capacity (NEAC), has been developed to assess the overall antioxidant activity from foods and beverages using different chemical assays, such as the ferric reducing antioxidant potential (FRAP), oxygen radical absorbance capacity (ORAC), Trolox equivalent antioxidant capacity (TEAC), vitamin C equivalent antioxidant capacity (VCEAC) and 2,2′-azino-bis (3-ethylbenzthiazoline) 6-sulfonic acid (ABTS), and the total radical-trapping antioxidant parameter (TRAP) (30).

### Data synthesis and statistical analyses

In this study, we used RRs and 95%CIs as the risk estimate for the main analysis. Also, we assumed that the HR was approximately equal to the RR (31). For the ORs, they were converted into RRs using the following formula: RR = OR/[(1-P_0_) + (P_0_*OR)], in which P_0_ shows the incidence of stroke in the non-exposed group (32). Log-transformed RRs and their corresponding standard errors (SEs) were obtained using risk ratios (full adjusted ORs, HRs and RRs and corresponding 95% CIs), which were previously extracted for the association between dietary TAC and stroke risk. We performed a pairwise meta-analysis by pooled the RRs and 95% CIs for the highest versus the lowest categories of dietary TAC in relation to the risk of stroke. Heterogeneity across studies was evaluated by the Cochran’s Q test and quantified by I^2^ statistics. In our analyses, if *p*-values of Cochran’s *Q* test <0.10 or *I*^2^ > 50% demonstrated the high heterogeneity, and a DerSimonnian and Laird random-effects model was used to pool the RRs. Conversely, a *p* value of *Q*-test >0.10 or *I*^2^ < 50% indicated an absence of heterogeneity among studies, and a fixed-effects model was used to calculate the pooled RRs (33). If the results indicated significant heterogeneity among studies, sensitivity and subgroup analyses were used to explore potential sources of heterogeneity. Subgroup analyses were performed based on study design (cohort or case–control studies), study region (Western countries or other), mean age (≥50 or <50), sample size (<5,000 or ≥5,000), study quality (≥7 or <7), and methods for dietary assessment (FFQ or 24 h dietary records). Sensitivity analysis was conducted to confirm whether the pooled RRs were robust or sensitive to the impact of a certain study. Publication bias was assessed via visual inspection of funnel plots and quantified by both Begg’s and Egger’s tests (34). If publication bias was found, the trim and fill method was used to re-calculate our results (35). Finally, we also performed a dose–response meta-analysis to estimate the trend from the correlated log RRs across the categories of dietary TAC scores. A two-stage GLST model based on generalized least squares was applied to examine the linear or non-linear dose–response relationship between dietary TAC and risk of stroke (36). We used dietary TAC modelling and restricted cubic splines with three knots at fixed percentiles (10, 50 and 90%) of the distribution. All statistical analyses were carried out using STATA/SE, version 12.0 (StataCorp, College Station, Texas, USA). We considered a *p-*value were ≤ 0.05 (two -sided) to be statistically significant unless otherwise specified.

## Results

### Overview of included studies for the systematic review

**Figure 1:**
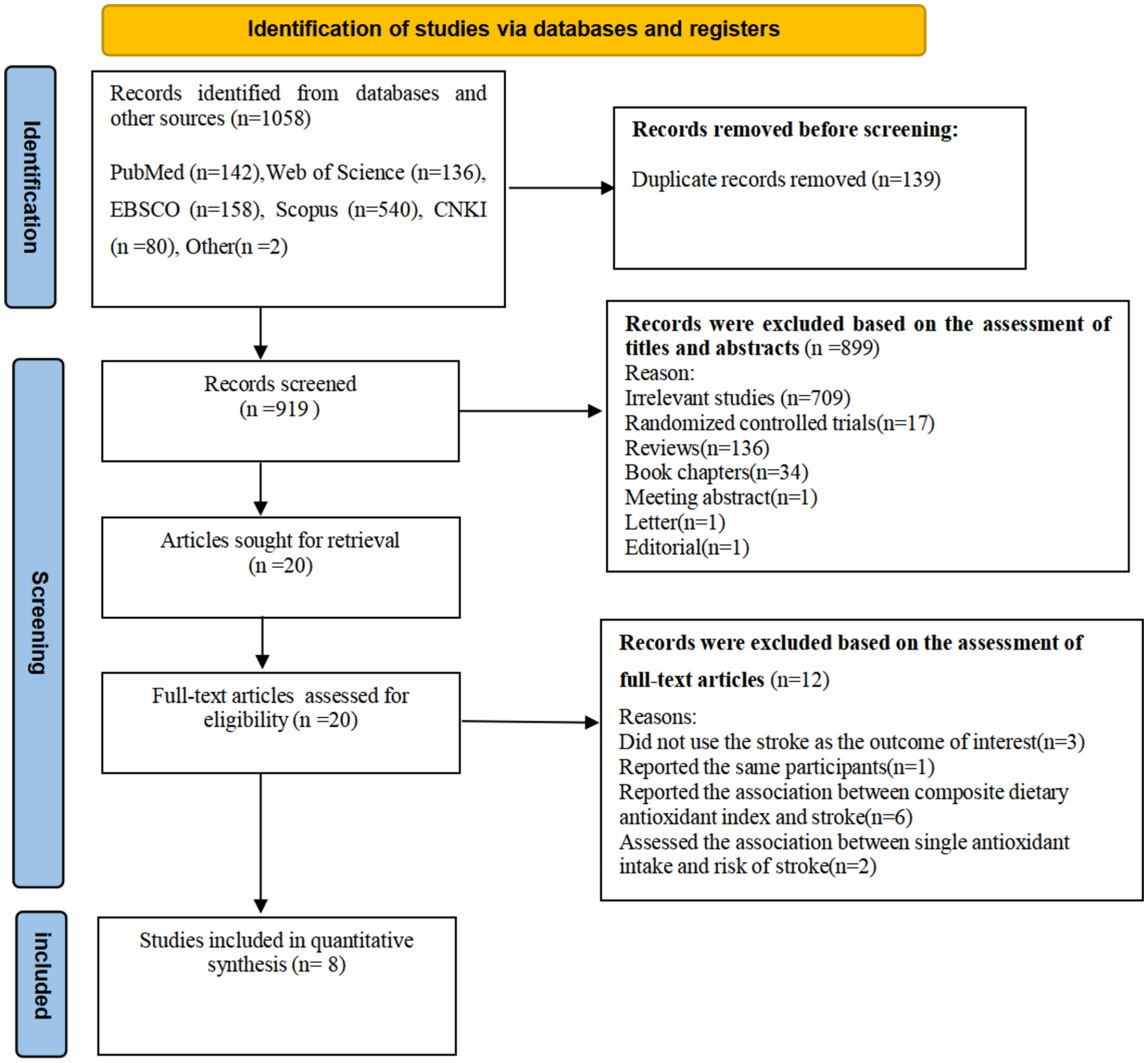
Flow chart of the process of the study selection.

The flow chart of literature search process is shown in Figure 1. In the initial literature search, a total of 1,058 relevant articles were retrieved for this study. After eliminating 139 duplicates, 919 articles were selected. Afterward, 901 articles were excluded based on the review of the titles and abstracts of retrieved articles. Eighteen full-text articles were independently reviewed in details. Out of the remaining 20 articles, 12 were excluded because of the following reasons: did not use the stroke as the outcome of interest (*n* = 3), reported the association between composite dietary antioxidant index and stroke (*n* = 6), assessed the association between single antioxidant intake and risk of stroke (*n* = 2), and reported the same participants (*n* = 1). Finally, eight articles were considered eligible for inclusion in this systematic review and meta-analysis (9, 20–26). The PICO for this meta-analysis is shown in Table 1.

**Table 1:** The PICOS criteria used for this systematic review and meta-analysis.

Population	Adults
Exposure	Dietary total antioxidant capacity
Comparison	Highest category vs. lowest category of exposure
Outcomes	Stroke
Study design	Observational studies with the design of cohort case–control or cross-sectional

PICOS, participant, intervention (exposure), comparison, outcome, and study design.

### Study characteristics

The characteristics of the eligible studies are listed in Table 2. A total of eight articles was included, with sample size of included studies ranging from 237 to 45,882. Of all the included studies, six were cohort studies (9, 22–26) and two were case–control studies (20, 21). With regards to the origin of the studies, three studies were carried out in Swedish (9, 23, 26), two in Iran (20, 21), one in the United States (22), one in Netherlands (23), and one in Italy (24). All of the included studies were published between 2011 and 2024. Two studies included women only (23, 26), and other six studies included both men and women (9, 20, 21, 24, 25). The follow-up duration for the cohort studies ranged from 7.9 to 20.2 years. The age of participants across studies ranged from ages 18 to above. Seven of included studies used FFQs to collect dietary data (9, 20, 21, 23–26), and one study used 24-h dietary recall (22). To measure the dietary TAC, five studies used the FRAP assay (9, 20, 21, 24, 26), one study used the ORAC assay (23), one study used VCEAC and ABTS assays (22), and one study used the TEAC assay (25). Based on the NOS, from eight included studies, seven studies were high quality (9, 21–26), and the remaining one study was medium quality (20). The quality assessment of included studies bases on NOS criteria is shown in Table 3.

**Table 2:** Characteristics of the included studies on the relationship between dietary total antioxidant capacity and risk of stroke.

Author Publication Year	Study region	Study design	Total number of participants	Age	Exposure assessment	Adjustment or matched for in the analyses	Outcomes
Colarusso et al. (2017) (9)	Sweden	Cohort	34,555 (1,186 cases)	≥20y	FFQ	Age, educational level, smoking status, body mass index, physical activity, self-reported hypertension, self-reported diabetes, aspirin use, dietary supplement use, coffee consumption, alcohol consumption, self-reported lipid disturbance, and total energy intake.	Highest Q4 vs. lowest Q1 of TAC (HR = 1.35, 95%CI:0.96–1.89); Q3 vs. Q1 (HR = 0.99, 95% CI:0.70–1.40); Q2 vs. Q1 (HR = 1.19, 95%CI: 0.85–1.67)
Habibzadeh et al. (2024) (20)	Iran	Case–control	237 (79 cases)	35-70y	FFQ	Energy intake, smoking, hypertension, hyperlipidemia, diabetes, and overweight	Tertile 3 vs. Tertile 1 of TAC (OR = 1.06,95% CI: 0.94–1.20);Tertile 2 vs. Tertile 1 of TAC (OR = 1.03, 95% CI: 0.93–1.15).
Milajerdi et al. (2020) (21)	Iran	Case–control	290 (195 cases)	≥20y	FFQ	Age, sex, energy intake, physical activity, smoking hypertension, diabetes, hyperlipidemia, obesity, dietary intake of fibers and omega-3 fatty acids	Tertile 3 vs. Tertile 1 of TAC (OR = 0.49, 95% CI: 0.23–1.00);Tertile 2 vs. Tertile 1 of TAC (OR = 0.88, 95% CI: 0.48–1.60).
Yang et al. (2022) (22)	United States	Cohort	37,045 (1,391 cases)	≥20y	24 h dietary records	Age, gender, race, alcohol, smoke, activity, diabetes, hypertension, dyslipidemia, cardiovascular disease, body mass index, neutrophil to lymphocyte ratio.	Highest tertile 3 vs. lowest tertile 1 of TAC (OR = 0.860, 95% CI: 0.726–1.019); Tertile 2 vs. tertile 1 of TAC (OR = 0.836, 95% CI: 0.706–0.990).
Rautiainen et al. (2022) (23)	Sweden	Cohort	31,035 (1,322 cases)	49–83y	FFQ	Age, education, smoking, body mass index, physical activity, hypertension, hypercholesterolemia, diabetes, family history of myocardial infarction, aspirin use, dietary supplement use, and intakes of total energy, alcohol, and coffee.	Highest quintile vs. lowest quintile of TAC (HR = 0.83, 95% CI: 0.70–0.99); quintile 4 vs. lowest quintile 1 of TAC (HR = 0.88, 95% CI: 0.74–1.04); quintile 3 vs. lowest quintile 1 of TAC (HR = 0.90, 95% CI: 0.76–1.07); quintile 2 vs. lowest quintile 1 of TAC (HR = 0.92, 95% CI: 0.78–1.09).
Devore et al. (2013) (24)	Netherlands	Cohort	5,395 (601 cases)	≥55y	FFQ	Age, total calorie intake, smoking, high blood pressure, diabetes, myocardial infarction, and supplement use.	Highest tertile 3 vs. lowest tertile 1 of TAC(RR = 0.91, 95% CI: 0.75–1.11);Highest tertile 2 vs. lowest tertile 1 of TAC(RR = 0.92, 95% CI: 0.76–1.13).
Del Rio et al. (2011) (25)	Italy	Cohort	41,620 (194 cases)	≥18y	FFQ	Hypertension, smoking status, education, non-alcohol energy intake, alcohol drinking, waist circumference, obesity, physical activity, center, sex, and age.	Highest tertile 3 vs. lowest tertile 1 of TAC (RR = 0.65, 95% CI: 0.41–1.02); Highest tertile 2 vs. lowest tertile 1 of TAC (RR = 0.97,95% CI: 0.67–1.41).
Hantikainen et al. (2020) (26)	Sweden	Cohort	45,882 (871 cases)	30–49y	FFQ	Age, education (≤10, 11–13, >13 y), body mass index (kg/m^2^), smoking (no, former, current), physical activity (low, medium, high), total alcohol intake(<5, 5–25, >25 g/d), total energy intake (kcal/d), multivitamin supplement use (yes, no), hypertension (yes, no), diabetes (yes, no), coffee intake (0, 0–4, >4 cups/d).	Highest quintile 5 vs. lowest quintile 1 of TAC (HR = 0.88, 95% CI: 0.68–1.12); quintile 4 vs. quintile 1 (HR = 0.93, 95% CI: 0.73–1.17); quintile 3 vs. quintile 1 of TAC (HR = 0.80, 95% CI: 0.63–1.01);quintile 2 vs. quintile1 of TAC (HR = 0.88, 95% CI: 0.70–1.10).

CI, confidence interval; FFQ, food frequency questionnaire; HR, Hazard ratio; OR, odds ratio; TAC, total antioxidant capacity.

**Table 3:** Subgroup analyses of stroke for the highest versus lowest category of dietary total antioxidant capacity intake.

Dietary total antioxidant capacity	Subgroup	No. of studies	RR (95%CI)	*P*-values	Heterogeneity		
					*P*-values for within groups	*I*^2^ (%)	*P-*values for between groups
Study design	Cohort	6	0.88 (0.81–0.96)	0.003	0.228	26.3	0.742
	Case–control	2	0.83 (0.61–1.13)	0.241	0.326	0.0	
Study region	Western countries	6	0.88 (0.81–0.96)	0.003	0.228	26.3	0.742
	Other	2	0.83 (0.61–1.13)	0.241	0.326	0.0	
Study quality	≥7	7	0.87 (0.80–0.95)	0.001	0.267	20.5	0.525
	<7	1	1.04 (0.61–1.78)	0.886	–	–	
Mean age	≥50y	6	0.89 (0.81–0.97)	0.011	0.252	23.2	0.454
	<50y	2	0.82 (0.67–0.99)	0.044	0.360	0.0	
Sample size	≥5,000	6	0.88 (0.81–0.96)	0.003	0.228	26.3	0.742
	<5,000	2	0.83 (0.61–1.13)	0.241	0.326	0.0	
Methods for dietary assessment	FFQ	7	0.88 (0.80–0.97)	0.008	0.242	23.6	0.815
	24 h dietary records	1	0.86 (0.73–1.02)	0.081	–	–	

CI, confidence interval; FFQ, food frequency questionnaire; RR, relative risk.

### Dietary TAC and stroke risk

**Figure 2:**
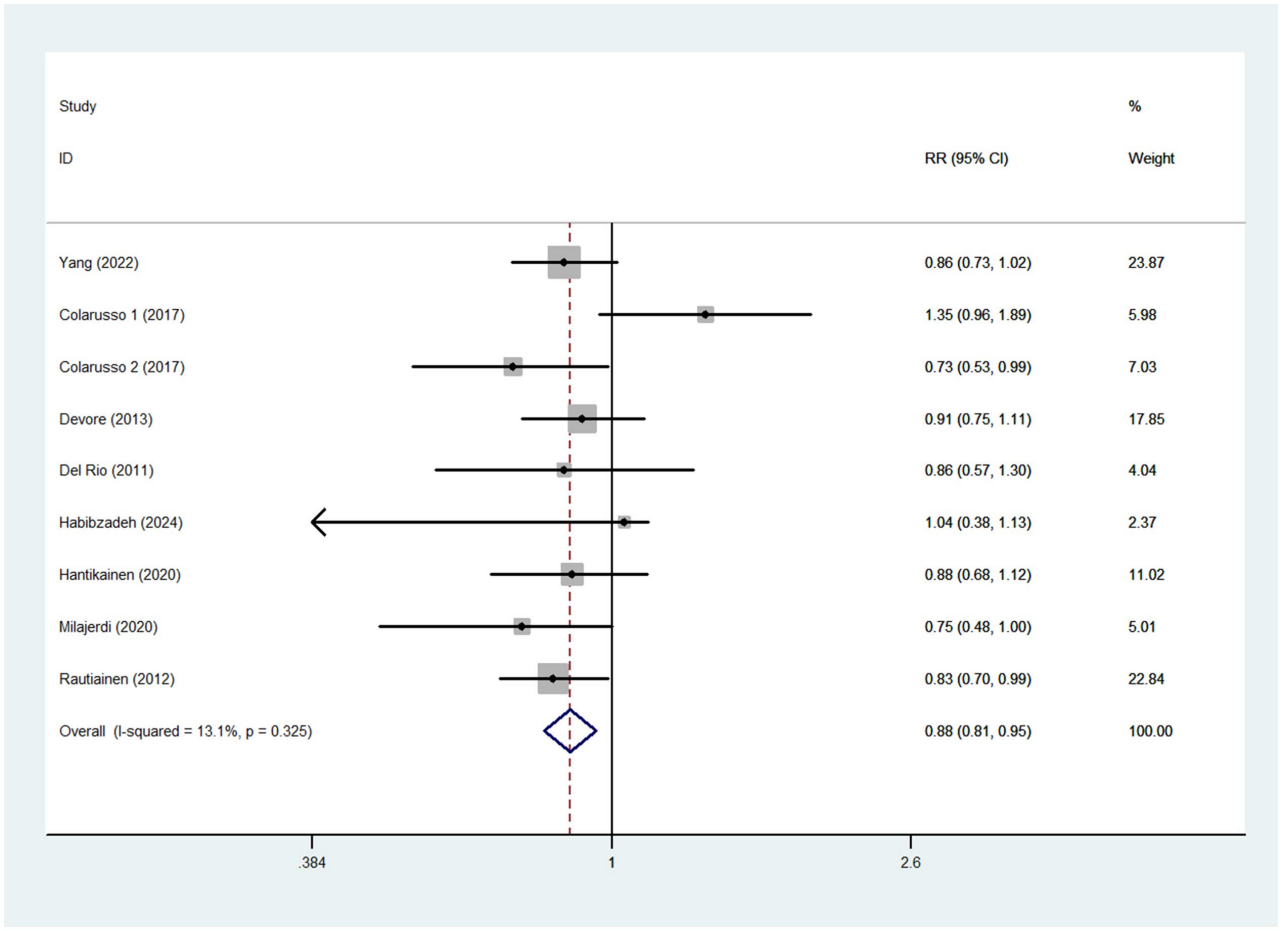
Forest plot of the association between dietary TAC intake and stroke risk.

Eight studies involving 196,059 participants and 5,839 stroke cases, were included to evaluate the association between dietary TAC and stroke risk. Combining nine effect sizes from eight studies, Figure 2 shows the evidence of a 12% lower risk of stroke in the highest compared with the lowest categories of dietary TAC scores (RR = 0.88; 95%CI: 0.81–0.95, *p* = 0.002). The low heterogeneity was observed in the included studies (*I*^2^ = 13.1%; *p* = 0.325), thus a fixed-effects model was used to calculate the pooled RRs. Given the low heterogeneity of this meta-analysis, subgroup analyses were not performed to explore the potential sources of heterogeneity across studies.

### Dose–response analysis

**Figure 3:**
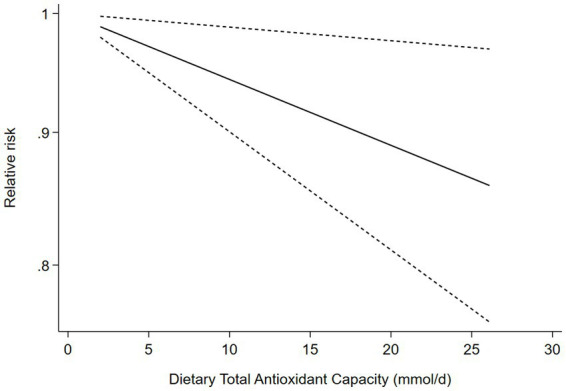
Dose-response association between dietary TAC and risk of stroke in the analysis of five cohort studies.

Five cohort studies (9, 23–26) were included in the dose–response analysis for the relationship between dietary TAC and risk of stroke (Figure 3). The dose–response meta-analysis indicated a linear association between dietary TAC and risk of stroke in the analysis of cohort studies (RR = 0.994; 95%CI:0.990–0.999, *P_dose–response_ =* 0.014, *P_non-linearity_ =* 0.329).

### Subgroup analyses

To further assess the probable sources of heterogeneity across included studies, we conducted subgroup analyses basing on study design, study region, mean age, sample size, study quality, and methods for dietary assessment (Table 3). The results of subgroup analyses showed that dietary TAC was statistically significant in the studies with mean age < 50 (RR = 0.82, 95%CI: 0.67–0.99, *p* = 0.044), and there was no evidence of heterogeneity (*p =* 0.360; *I*^2^ = 0.0%). Moreover, there was an inverse association between dietary TAC intake and risk of stroke in cohort studies, Western countries and sample size≥5,000 (RR = 0.88; 95%CI: 0.81–0.96, *p* = 0.003), with less evidence of heterogeneity (*p =* 0.228; *I*^2^ = 26.3%).

### Publication bias

As shown in Supplementary Figure S1, inspection of funnel plots revealed little evidence of asymmetry. Begg’s test for publication bias had no statistical significance (highest compared with lowest categories of dietary TAC: *p* = 0.602). In addition, Egger’s test for publication bias had no statistical significance (*p* = 0.559).

### Sensitivity analysis

In sensitivity analysis (Supplementary Figure S2), the results showed that the association between dietary TAC and stroke risk was robust and not affected by any single study or a couple of studies.

### Quality assessment

The quality of included studies using NOS criteria is shown in Table 4. Seven out of eight included studies received NOS scores ≥7 points, and were classified as of high- quality (9, 21–26). In addition, the remaining one article was classified as of medium-quality (20).

**Table 4:** Dietary total antioxidant capacity and risk of stroke: assessment of study quality.

Studies	Selection				Comparability		Outcome			Score
	1	2	3	4	5A	5B	6	7	8	
Cohort										
Colarusso et al. (2017) (9)	*	*	*	*	*	*	*	*	*	9
Yang et al. (2022)(22)	*	*	*	*	*		*	*	*	8
Rautiainen et al. (2012) (23)	*	*	*	*	*	*	*	*	*	9
Devore et al. (2013) (24)	*	*	*	*	*	*	*	*	*	9
Del Rio et al. (2011) (25)	*	*	*	*	*		*	*	*	8
Hantikainen et al. (2020) (26)	*	*	*		*	*	*	*		7
Case–control										
Habibzadeh et al. (2024) (20)	*	*	*		*		*	*		6
Milajerdi et al. (2020) (21)	*	*	*		*	*	*	*		7

*For case–control studies, 1 indicates cases independently validated; 2, cases are representative of population; 3, community controls; 4, controls have no history of CKD; 5A, study controls for the most important factor; 5B, study controls for additional factor(s), e.g., cigarette smoking body mass index, total energy intake; 6, ascertainment of exposure by blinded interview or record; 7, same method of ascertainment used for cases and controls; and 8, non-response rate the same for cases and controls. For cohort studies, 1 indicates exposed cohort truly representative; 2, non-exposed cohort drawn from the same community; 3, ascertainment of exposure by secure record (e.g., surgical records) or structured interview; 4, outcome of interest was not present at start of study; 5A, study controls for the most important factor; 5B, study controls for additional factor(s); 6, assessment of outcome is based on independent blind assessment or record linkage; 7, follow-up long enough (≥5 years) for outcomes to occur; and 8, adequacy of follow up of cohorts (all participants complete follow up or > 90% participants complete follow up).

## Discussion

As far as we know, this study is the first systematic review and dose–response meta-analysis to exclusively and systematically evaluate the association between dietary TAC and risk of stroke. The results showed that higher dietary TAC was associated with a reduced risk of stroke. Additionally, the dose–response meta-analysis revealed a significant linear association between dietary TAC and risk of stroke in cohort studies. Sensitivity analysis showed that the pooled results were robust and not affected by any single study or a couple of studies. Our findings corroborate previous research and underscore the important role of dietary TAC in the prevention of stroke.

Although the incidence of stroke has decreased in some Western countries, it remains as the second leading cause of death worldwide (20). Given the global public health concern, it is crucial to clarify effective prevention strategies for stroke. Dietary factors have consistently been recognized as an important and modifiable risk factors for stroke (37). Notably, previous epidemiological studies have primarily focused on the intake of single antioxidants or a group of antioxidants in relation to stroke risk, yielding inconsistent findings (12, 13). However, little is known with regard to the association between dietary TAC intake and risk of stroke. Dietary TAC measures the overall antioxidant capacity of a diet, considering the synergistic effects and interactions of various antioxidants (16). As mentioned above, several epidemiological studies have examined the relationship between dietary TAC and risk of stroke (9, 20–26). But, findings from previously published studies remain inconclusive. For instance, Habibzadeh et al. in a recent nested case–control study, found no significant association between dietary TAC and stroke risk (20). Conversely, a Swedish cohort study of 36, 715 women by Rautiainen et al., showed that dietary TAC was inversely associated with total stroke among cardiovascular disease (CVD)-free women and hemorrhagic stroke among women with CVD history (23). Similarly, our current study found that higher dietary TAC intake was associated with a lower risk of stroke in the current study. The discrepant findings in previous studies May be attributed the following several factors. First, the methods for assessing dietary TAC are different. For example, Yang et al., used vitamin C equivalent antioxidant capacity and ABTS assay to determine the dietary TAC (22), while Del Rio et al., used the TEAC assay in a large Italian cohort (25). Other studies used the FRAP assay (9, 20, 21, 24, 26), which might underestimate the true antioxidant capacity of the whole diet by not considering lipophilic antioxidant (38). Second, discrepancies in dietary data collection methods May contribute to the differing results. Yang et al. used 24-h dietary recalls to evaluate the usual dietary intake (22), whereas the remaining seven studies used a FFQ (9, 20, 21, 23–26). Third, there were the significant differences in dietary habits and lifestyle between different countries. Six of the included studies were conducted in Western countries (9, 22–26), and the remaining two studies in Iran (20, 21). Notably, there are stark differences between Iranian and Western diets. Fourth, the types of foods that contribute the most to dietary TAC vary widely in different countries. In short, discrepancies in the measurement of dietary TAC, types of dietary questionnaire, and differences in dietary habits and lifestyles across countries May contribute to the different results.

Even though evidence on the association between dietary TAC and risk of stroke is inconsistent, several mechanisms have been proposed to explain the observed favorable associations. First, previous studies have indicated that antioxidants abundant in fruits and vegetables can help to prevent oxidative stress (39), which plays a critical role in the pathogenesis of stroke (40). Antioxidants are compounds that scavenge reactive oxygen species (ROS) and protect cells from oxidative damage (41). Second, antioxidants can improve endothelial dysfunction and lower blood pressure, both of which are the known risk factors for stroke (42, 43). A recent systematic review and meta-analysis reported that higher intake of dietary TAC was associated with reduced systolic blood pressure, diastolic blood pressure, and fasting blood sugar, all factors associated with the risk of stroke (4, 30). Third, dietary TAC intake has been inversely associated with plasma concentrations of C-reactive protein, a sensitive biomarker of systemic inflammation (44). Systemic inflammation is a well-known risk factor in the etiology and pathology of stroke (45). All together, these mechanisms discussed above May explain the protective effect of high dietary TAC intake on stroke risk.

### Strengths and limitations of study

There are some strengths and limitations in this meta-analysis. First, this is the first systematic review and dose–response meta-analysis thus far assessing the association between dietary TAC and risk of stroke. Our findings add the evidence for a protective effect of high dietary TAC on stroke, and highlight the importance of increasing dietary TAC intake for the prevention of stroke. Second, stroke diagnosis was confirmed through medical records, minimizing misdiagnosis bias. Third, no signs of publication bias were evident in the funnel plot, and statistical tests for publication bias were also non-significant. Fourth, rigorous article selection was made according to pre-determined inclusion and exclusion criteria. Finally, the dose–response analysis was also conducted to strengthen the association between dietary TAC and stroke risk. Despite the aforementioned strengths, several limitations should also be considered in this study. First, given the observational nature of all included studies, the possibility of residual bias remains. In parallel, two of included studies were case–control studies, which have inherent limitations of recall and selection bias. Thus, we cannot assume the causality of the observed association. Therefore, further prospective cohort studies or randomized controlled trials are needed to confirm the role of dietary TAC in the prevention of stroke. Second, in the present study, dietary TAC measurement was calculated based on self-reported data gathered by 24 h dietary records and FFQs, which might cause misclassification, thereby resulting in the under-or overestimation of dietary TAC. Third, although multiple potential confounding variables have been taken into account, the existence of residual confounders cannot be excluded owing to the undetected or unknown confounders. In addition, adjusted confounding factors were inconsistent across all included studies, leading to some degree of variation in the values of OR, RR or HR. Finally, six of the included studies were performed in Western countries, with only two studies in Asian countries, limiting the generalizability of our findings.

## Conclusion

In conclusion, our study demonstrated that higher intake of dietary TAC was significantly associated with a reduced risk of stroke. Our findings contribute additional evidence supporting the favorable effect of high dietary TAC on stroke, and highlight the importance of promoting consumption of dietary TAC for the prevention of stroke. Additionally, it also makes sense to elucidate the potential association between dietary TAC and stroke risk and provides a scientific basis for developing dietary guidelines. Future well-designed prospective studies, particularly in diverse geographic regions and settings, are needed to confirm these findings.

## Data Availability

The original contributions presented in the study are included in the article/Supplementary material, further inquiries can be directed to the corresponding author.
