# Supplementary figures and images for: Dietary total antioxidant capacity and risk of stroke: a systematic review and dose–response meta-analysis of observational studies
Source: Front Nutr. 2024 Sep 19;11:1451386. doi: 10.3389/fnut.2024.1451386 (PMC11448356; doi:10.3389/fnut.2024.1451386)

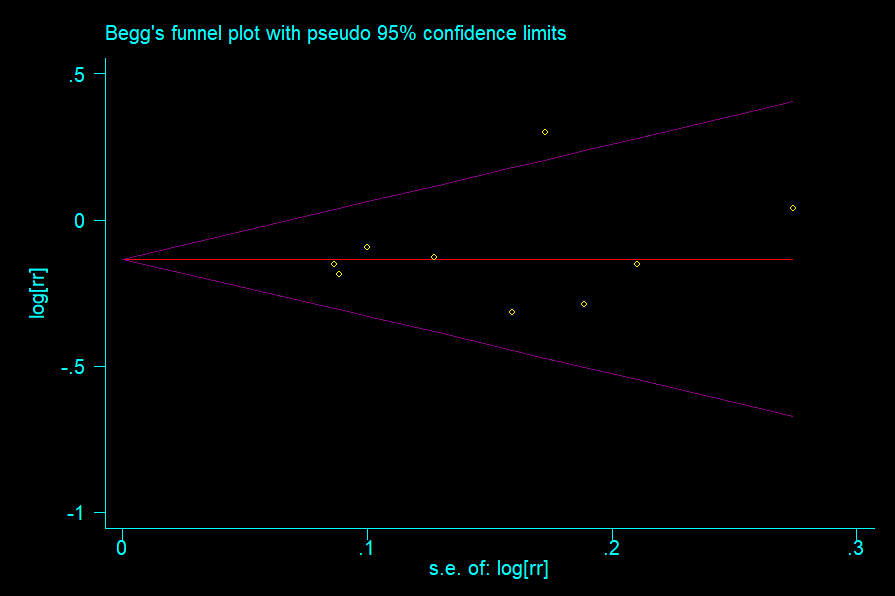

Supplement: Supplementary file 1 [file Image_1.TIF]

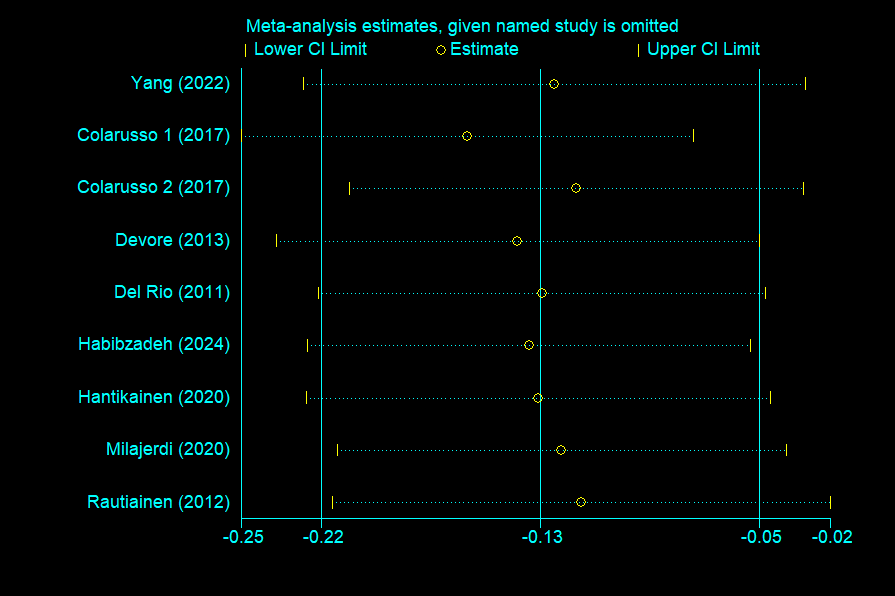

Supplement: Supplementary file 2 [file Image_2.TIF]
